# Renal temperature reduction progressively favors mitochondrial ROS production over respiration in hypothermic kidney preservation

**DOI:** 10.1186/s12967-019-2013-1

**Published:** 2019-08-13

**Authors:** Koen D. W. Hendriks, Isabel M. A. Brüggenwirth, Hanno Maassen, Albert Gerding, Barbara Bakker, Robert J. Porte, Robert H. Henning, Henri G. D. Leuvenink

**Affiliations:** 1 Department of Clinical Pharmacy and Pharmacology, University Medical Center Groningen, University of Groningen, Hanzeplein 1, 9713JZ Groningen, The Netherlands; 2 Department of Surgery, University Medical Center Groningen, Groningen, The Netherlands; 3 Section of Hepatobiliary Surgery and Liver Transplantation, Department of Surgery, University Medical Center Groningen, University of Groningen, Groningen, The Netherlands; 4 Department of Laboratory Medicine, University Medical Center Groningen, Groningen, The Netherlands; 5 Department of Pediatrics, University Medical Center Groningen, Groningen, The Netherlands

**Keywords:** Machine perfusion, Hypothermic preservation, Kidney transplantation, Reactive oxygen species, Mitochondrial function

## Abstract

**Background:**

Hypothermia, leading to mitochondrial inhibition, is widely used to reduce ischemic injury during kidney preservation. However, the exact effect of hypothermic kidney preservation on mitochondrial function remains unclear.

**Methods:**

We evaluated mitochondrial function [i.e. oxygen consumption and production of reactive oxygen species (ROS)] in different models (porcine kidney perfusion, isolated kidney mitochondria, and HEK293 cells) at temperatures ranging 7–37 °C.

**Results:**

Lowering temperature in perfused kidneys and isolated mitochondria resulted in a rapid decrease in oxygen consumption (65% at 27 °C versus 20% at 7 °C compared to normothermic). Decreased oxygen consumption at lower temperatures was accompanied by a reduction in mitochondrial ROS production, albeit markedly less pronounced and amounting only 50% of normothermic values at 7 °C. Consequently, malondialdehyde (a marker of ROS-induced lipid peroxidation) accumulated in cold stored kidneys. Similarly, low temperature incubation of kidney cells increased lipid peroxidation, which is due to a loss of ROS scavenging in the cold.

**Conclusions:**

Lowering of temperature highly affects mitochondrial function, resulting in a progressive discrepancy between the lowering of mitochondrial respiration and their production of ROS, explaining the deleterious effects of hypothermia in transplantation procedures. These results highlight the necessity to develop novel strategies to decrease the formation of ROS during hypothermic organ preservation.

**Electronic supplementary material:**

The online version of this article (10.1186/s12967-019-2013-1) contains supplementary material, which is available to authorized users.

## Background

The current shortage of donor organs remains a major problem in transplantation medicine. To expand the donor pool, an increasing number of suboptimal organs are accepted [1, 2]. These suboptimal, or so-called extended criteria donor organs, are more susceptible to the ischemic periods during procurement, preservation, and eventually transplantation [3]. After transplantation of the organ in the recipient, the pre-existing injury to cells, together with the ensuing influx of immune cells, results in substantial organ injury, a phenomenon known as ischemia/reperfusion (I/R) injury [3]. Key to I/R injury is mitochondrial failure, both resulting in reduction of adenosine triphosphate (ATP) production and the generation of reactive oxygen species (ROS) [4, 5]. Nowadays, several techniques are employed to mitigate organ damage, such as the inclusion of antioxidants in preservation solutions and the use of oxygenated hypothermic machine perfusion (HMP), which both reduce the formation of ROS and improve post-transplantation graft function [5–8]. Nevertheless, cooling, either by static cold storage or HMP, remains the cornerstone in organ preservation, and has been widely accepted and successfully used for decades. The beneficial effects of forced cooling are attributed to the deceleration of all metabolic processes, resulting in a hypometabolic state with decreased mitochondrial activity as reflected by lower oxygen consumption [9].

It should be noted, however, that a kidney undergoes various temperature shifts during the course of a transplantation procedure. Immediately after procurement, the core organ temperature is around 33 °C. After the first cold flush in the donor, temperature drops to 10–20 °C, decreasing further to 1–3 °C during subsequent cold storage. During backtable preparations in preparation for transplantation, temperature increases slightly to 2–6 °C with a rapid increase to around 20 °C just before reperfusion in the recipient procedure and a subsequent full rewarming once vascular anastomoses are in place [10, 11].

To our knowledge, the exact effect of cooling at different temperatures on kidney mitochondrial oxygen consumption and ROS production are hitherto unreported. A better understanding of mitochondrial behavior at different temperatures may yield information that is critical to optimize transplantation protocols and reduce I/R injury. Here, we explored mitochondrial oxygen consumption in porcine kidneys while mimicking the transplantation procedure by manipulating temperature in a closed kidney perfusion circuit. To verify results obtained in whole kidney and identify the underlying mechanisms, we additionally examined mitochondrial function, ROS production and scavenging-capacity in isolated mitochondria and in an in vitro cell model.

## Methods

### Kidney procurement

Porcine kidneys were obtained from a local slaughterhouse. Pigs averaging 5 months of age and 130 kg were killed by electrocution followed by exsanguination. After 30 min of circulatory arrest, kidneys were obtained, flushed via the renal artery with 180 ml of ice-cold NaCl 0.9% (Baxter BV, Utrecht, The Netherlands) and subsequent HMP was applied.

### Hypothermic machine perfusion

HMP (Kidney Assist Transport, Organ Assist, Groningen, The Netherlands) was used for preservation of the kidney, delivering a pulsatile flow of Belzer University of Wisconsin (UW) machine preservation solution (Belzers MP, Bridge to Life Ltd., London) at 4 °C oxygenated with 100% O_2_ at a rate of 100 ml/min. The system was pressure-controlled with a mean arterial pressure of 25 mmHg.

### Closed-circuit kidney perfusion at different temperatures

After 2 h of HMP, kidneys (n = 4) were flushed with 50 ml of cold NaCl 0.9%, placed in an organ chamber and connected to a closed-circuit kidney perfusion system with a mean arterial pressure of 80 mmHg. The system was perfused with 500 ml Williams Medium E (Life technologies, USA) supplemented with 400 mg/l amoxicillin–clavulanate (Sandoz, Almere, The Netherlands), 0.112 mg/l creatinine (Sigma-Aldrich, The Netherlands) and 80 g/l Bovine Serum Albumin (PAA Laboratories GmbH, Austria). The oxygenator was supplied with a carbogen mixture of 95% O_2_ and 5% CO_2_ at a flow of 500 ml/min. Temperature of the perfusion fluid was controlled by a water bath connected to the oxygenator throughout the experiment. Arterial and venous oxygenous tensions as well as kidney flow were continuously recorded. Oxygen consumption was calculated as ΔhPa (pO_2_ [hPa] arterial − pO_2_ venous [hPa]) * (flow [ml/min]/weight [g]).

The initial temperature of the perfusion solution was set at 7 °C for 15 min. Subsequently, temperature was increased by 5 °C every 15 min until a temperature of 37 °C was reached. Also, the decreasing curves was evaluated: after the increasing temperature curve, the same kidneys were exposed to similar measurements while decreasing the temperature every 15 min from 37 to 7 °C.

#### Oxygen consumption in isolated porcine kidney mitochondria

Porcine kidney biopsies (n = 5) were obtained within 30 min after circulatory death of the animal and stored on ice for maximal 3 h in a mitochondrial buffer solution (250 mM sucrose, 10 mM TRIS and a pH of 7.4 at 4 °C). Then, mitochondria were isolated according to the method of Mildaziene et al. [12] and placed on ice.

Mitochondrial oxygen consumption was measured using a Clark electrode (Rank Brothers Ltd, UK), with the chamber of the oxygraph connected to a water bath, enabling adjustment of temperature between 7 and 37 °C. After a stable temperature was reached, stored mitochondria were suspended in 800 μl of Mitochondrial Respirometry Solution (MiR05: 0.5 mM EGTA, 3 mM MgCl_2_·6H_2_O, 20 mM taurine, 10 mM KH_2_PO_4_, 20 mM HEPES, 1 g/l BSA, 60 mM potassium-lactobionate, 110 mM sucrose, pH 7.1 at 30 °C). State three respiration was measured in response to the substrates glutamate and malate with an ADP generating system consisting of ATP (500 mM), hexokinase (500 U/ml), and glucose (1 M). Oxygen consumption rates were normalized to protein content (Bradford assay, Bio-Rad) and expressed as pmol O_2_/min/mg mitochondrial protein. A Q_10_ line was fitted for both the increasing en decreasing curve for each kidney, using R (version 3.5.1) package respirometry.

#### Mitochondrial H_2_O_2_ production in isolated porcine kidney mitochondria

Porcine kidney mitochondria were isolated as described above and energized with succinate in a mitochondrial buffer (20 mM MOPS, 110 mM KCl, 10 mM ATP, 10 mM MgCl_2_, 10 mM sodium succinate and 1 mM EGTA pH 7.5). H_2_O_2_ production was measured by Amplex red assay (ThermoFisher, USA) at 4, 22 and 37 °C every 10 min for 30 min by measuring fluorescence (585 nM using a Synergy 2 Multi-Mode plate reader, BioTek). Fluorescence levels were corrected for mitochondrial protein and expressed as relative values to its 37 °C value.

#### Lipid peroxidation in cold stored porcine kidneys

Porcine kidneys were obtained as described above (n = 4) and preserved on ice (4 °C) in UW solution for 16 h. Lipid peroxidation was quantified by measurement of malondialdehyde (MDA) using the OxiSelect TBARS assay kit (Cell Biolabs, USA) in samples taken 30 min after slaughter (37 °C) and after 16 h of cold storage. Lipid peroxidation levels were corrected for the amount of protein and expressed as relative to normothermic levels.

#### Cell culture

Human epithelial kidney cells (HEK293) were cultured in Dulbecco’s modified eagle medium (Gibco, USA) supplemented with 10% fetal bovine serum (Gibco, USA) and 1% penicillin/streptomycin. For experiments, cells were plated in poly-l-lysine coated 6 wells plates and grown until 90% confluency.

#### Oxygen consumption in HEK293 mitochondria

HEK293 cells were freshly trypsinized and resuspended in Hank’s balanced salt solution (Gibco, USA) supplemented with 25 mM HEPES. Oxygen consumption was measured as described above, using 1.5 × 10^6^ cells per experiment. Cells were permeabilized using digitonin (20 μg/ml) to allow entrance of substrates. Uncoupled respiration was measured in the presence of FCCP (1 μM, Sigma-Aldrich). Oxygen consumption was expressed as percentage of its 37 °C value.

#### Mitochondrial membrane potential

HEK293 cells were plated in 96 wells dark plates and incubated at 37 °C or 4 °C. Mitochondrial membrane potential was measured using JC1 (1 μg/ml, Sigma-Aldrich) by quantifying the fluorescence emission shift from green (529 nm) monomers to red (590 nm) aggregates. FCCP (1 μM, Sigma-Aldrich) was used as uncoupled control. Data are expressed as ratio red/green, relative to control at 37 °C.

#### Lipid peroxidation in HEK293 cells

HEK293 cells were incubated for 6 h at different temperatures (7, 17, 22, 32, 37 °C) or treated with 500 μM H_2_O_2_ for 4 h at 37 °C or 4 °C. After harvesting, lipid peroxidation was quantified as described above and expressed relative to 37 °C.

#### Mitochondrial superoxides

HEK293 cells were cultures in sterile 96 wells dark plates till confluent. 1 h prior the experiment, cells were washed with pre-warmed HBSS and medium was replaced with HBSS. At the same time, for the positive control, antimycin A was added to the positive controls. Afterwards, MitoSox reagent was added (5 μM, Thermofisher) and plates were incubated for 90 min at 37, 22 or 4 °C. Fluorescence was measured (ex/em: 510/580 nm) using a Synergy 2 Multi-Mode plate reader (BioTek).

#### Cell survival assay

Cell viability was assessed by a Neutral Red (NR) assay to quantify the number of living cells. Following incubation with 50 mg/ml NR dye (Sigma Aldrich) for 1 h at 37 °C, cells were lysed, and absorbance was measured at 450 nm using a Synergy 2 Multi-Mode plate reader (BioTek).

#### Western blotting

Cell lysates were obtained using RIPA lysis buffer (50 mM Tris–Cl pH = 8.0, 150 mM NaCl, 1% Igepal Ca 630, 0.5% Sodium Deoxycholate, 1.0% SDS, 0,4% protein inhibitor cocktail, 1 mM sodium orthovanadate, 10 mM NaF, 10 mM B-mercaptoethanol). Protein concentration was measured with a Bio-Rad protein assay on a Synergy H4 plate reader. Samples were loaded to 4–20% SDS pre-casted gels (Bio-Rad TGX gels) and transferred to a nitrocellulose membrane (Bio-Rad). The membranes were blocked with 5% skimmed milk and incubated with primary (O/N at 4  °C) and secondary (1 h at room temperature) and visualised using SuperSignal (Perkin Elmer) on a ChemiDoc MP imaging system (Bio-Rad) and quantified using ImageLab 6.0 (Bio-Rad). Antibodies used were: anti-MnSOD2 (1:1.000 Enzo), anti-B-actin (1:10.000 Santa Cruz), Goat-anti-rabbit-peroxidase (GARPO, 1:000 Dako). Full membranes are shown in Additional file 1: Figure S1.

### Statistical methods

Values were presented as mean ± standard error of the mean (SEM). Statistical analysis was performed using SPSS Statistics 23. Graphs were made using GraphPad PRISM version 2.0 software (San Diego, CA). Graphs and Q_10_ line presented in Additional file 1: Figure S2 were made in R (version 3.5).

## Results

### Lowering temperature dramatically decreases mitochondrial oxygen consumption, in accord of a Q_10_ effect

**Fig. 1:**
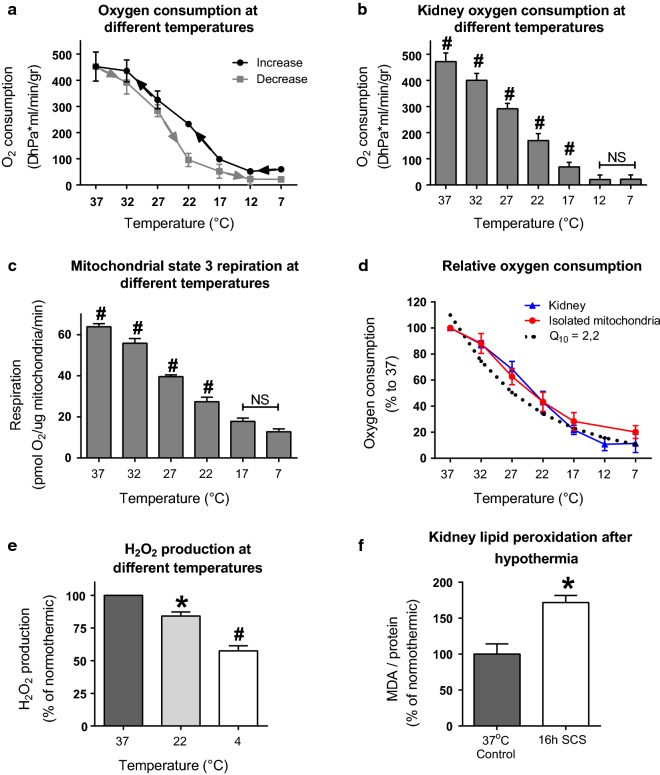
Temperature effects on porcine kidneys. **a** Oxygen consumption [expressed as ΔhPa (ml/min/gr)] in perfused porcine kidneys. The increasing and decreasing curve are expressed. Based on four independent experiments, expressed as mean, error bars represent SEM. **b** Average of whole kidney oxygen consumption per temperature. Expressed as mean, error bars represent SEM. ^#^Significant (ANOVA with Bonferroni posthoc, p < 0.001). **c** State three respiration in isolated mitochondria, in response to glutamate and malate with and an ADP generating system. Based on five independent experiments, expressed as mean, error bars represent SEM. ^#^Significant (ANOVA with Bonferroni posthoc, p < 0.001). **d** Oxygen consumption relative to 37 °C for perfused kidneys and isolated kidney mitochondria. The theoretical Q_10_ line is plotted (Q_10_ = 2). **e** Relative ROS production, measured as mitochondrial H_2_O_2_ production at different temperatures in isolated mitochondria from porcine kidneys. N = 4, expressed as percentage of its normothermic control, error bars represent SEM. *p < 0.01, ^#^p < 0.001 (ANOVA with Bonferroni posthoc). **f** ROS damage, measured as lipid peroxidation in porcine kidneys before and after 16 h of static cold storage in UW at 4 °C. N = 4, expressed as relative to normothermic, error bars represent SEM. *p < 0.01, (Students t test)

To assess the influence of temperature, whole kidney oxygen consumption was measured in a closed kidney perfusion model at different temperatures, both in an increasing and decreasing curve (Fig. 1a). Increasing and decreasing showed very similar results, however, a shift was seen around 22 °C. The increasing curve showed higher oxygen consumption compared to de decreasing curve. The average of the increasing and decreasing curve is plotted in Fig. 1b. Lowering of temperature from 37 °C to just below room temperature, 17 °C, provoked a strong decrease in oxygen consumption, with oxygen consumption stabilizing at temperatures below 17 °C. Live oxygen and flow registrations are shown in Additional file 1: Figure S1A and B.

To substantiate that the cold induced decrease in renal oxygen consumption originates from inhibition of mitochondrial respiration, we isolated porcine kidney mitochondria and measured state three (mitochondria with unlimited access to substrates, indicating maximal respiration) oxygen consumption at different temperatures (Fig. 1c). When expressed as relative to 37 °C, oxygen consumption decreased similarly in isolated mitochondria compared to whole kidney (Fig. 1d). Q_10_ reflects the rate of change of a physiological process as a consequence of a 10 °C change in temperature. Whereas a Q_10_ around 2 is typical for a strict chemical reaction, higher Q_10_ factors reflect involvement of biological processes. Interestingly, the observed oxygen consumption in the present study has a Q_10_ of 2.2 (as fitted in Fig. 1d), advocating that temperature affects a chemical (single enzymes) rather than a biological process. In Additional file 1: Figure S2, Q_10_ lines were plotted for every kidney perfusion experiment for both the increasing and decreasing temperature route.

### Lowering temperature induces ROS damage due to only modest inhibition of mitochondrial H_2_O_2_ production

To investigate whether decreased mitochondrial oxygen consumption at lower temperatures is accompanied by decreased ROS production, mitochondrial H_2_O_2_ production was measured at different temperatures in isolated mitochondria from porcine kidneys using the Amplex Red assay. Validation of temperature effects of the assay are presented in Additional file 1: Figure S2A. Cooling at 4 °C lowered mitochondrial H_2_O_2_ production by about 50% compared to 37 °C (Fig. 1e), signifying that the decrease in H_2_O_2_ was relatively smaller compared to the decrease in oxygen consumption. To substantiate that ROS-induced damage accumulates at lower temperatures, we measured MDA in porcine kidneys that were cold stored at 4 °C for 16 h. Indeed, MDA increased nearly twofold when compared to 37 °C (p < 0.01, Fig. 1f).

### Hypothermia induces failure of endogenous antioxidant capacity

**Fig. 2:**
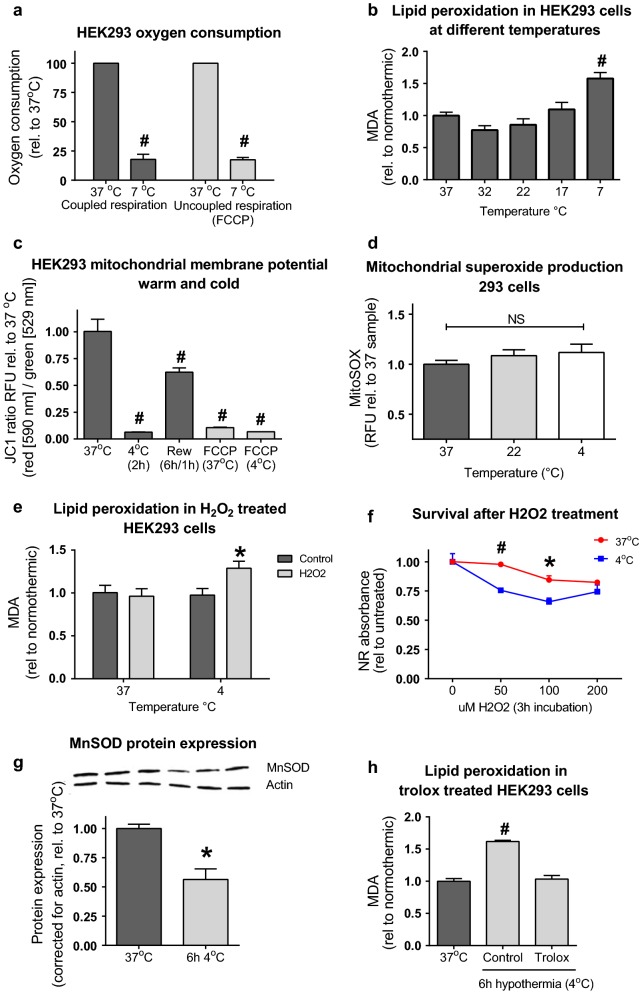
Temperature effects on HEK293 cells. **a** State three oxygen consumption at normothermic (37 °C) and hypothermic (4 °C) circumstances in coupled and uncoupled HEK293 cells. Based on three independent experiments, expressed as rel. to normothermic, error bars represent SEM. ^#^p < 0.001 (Students T test). **b** Lipid peroxidation (MDA) at different temperatures in HEK293 cells. N = 6, expressed as MDA levels rel. to normothermic, error bars represent SEM. ^#^p < 0.001 compared to 37 °C (ANOVA with Bonferroni posthoc). **c** Mitochondrial membrane potential in normothermic, hypothermic and rewarmed (rew) HEK293 cells. N = 6, expressed as JC1 ratio RFU rel. to 37 °C (red [590 nm]/green [529 nm]), error bars represent SEM. ^#^p < 0.001 compared to 37 °C (ANOVA with Bonferroni posthoc). FCCP (carbonyl cyanide 4-(trifluoromethoxy)phenylhydrazone 1 μM) as uncoupled control. **d** Mitochondrial superoxide production in HEK293 cells, incubated for 90 min at 37°, 22° or 4°. N = 6, expressed as mitosox RFU, error bars represent SEM. ^#^p < 0.001 compared to 37 °C (ANOVA with Bonferroni posthoc). **e** Lipid peroxidation in H_2_O_2_ stimulated normothermic and hypothermic treated HEK293 cells. N = 6, expressed as MDA levels rel. to normothermic, error bars represent SEM. *p < 0.05 compared to control (Student t test). **f** HEK293 survival after 3 h H_2_O_2_ exposure at different concentrations and temperatures. N = 3, expressed as Neutral Red absorbance rel. to untreated control. *p < 0.05 compared to non-treated (Student t test), ^#^p < 0.001 (Students T test). **g** MnSOD protein expression in normothermic and 6 h hypothermic (4 °C) HEK293 cells. Expressed as corrected values for actin, rel. to 37 °C, error bars represent SEM. *p < 0.05 (Student t test). Full membranes shown in Additional file 1: Figure S3B. **h** MDA levels in trolox treated 6 h hypothermic HEK293 cells. N = 3, expressed as MDA levels rel. to normothermic, error bars represent SEM. ^#^p < 0.001 compared to 37 °C (ANOVA with Bonferroni posthoc)

To further explore the effects of hypothermia on a deeper level, we used kidney HEK293 cells. To verify that cooling affects HEK293 similarly to whole organ, HEK293 oxygen consumption and lipid peroxidation were measured at different temperatures. Similar to porcine whole kidney, cooling at 7 °C induced reduction of oxygen consumption and increase of MDA in cells (Fig. 2a, b). Additionally, we measured mitochondrial membrane potential in normothermic, hypothermic and rewarmed HEK293 cells (Fig. 2c). In line with decreased oxygen consumption, mitochondrial membrane potential decreases strongly in hypothermia, which was partly restored after rewarming. Mitochondrial superoxide production was measured using a Mitosox assay. Cooling did not induce a decrease in superoxide formation (Fig. 2d). Together, these results indicate that cooling also produces a relative smaller decrease in ROS production than oxygen consumption in HEK293 cells, comparable to whole kidneys.

Next, we hypothesized cooling to decrease the overall anti-oxidant capacity resulting in increased MDA levels at low temperatures. To explore this, we challenged both normothermic and cooled HEK293 with an exogenous oxidant (H_2_O_2_) and measured MDA levels. Normothermic HEK293 cells were able to scavenge H_2_O_2_ without increased MDA levels (Fig. 2e). In contrast, cold incubated HEK293 cells showed a clear increase in MDA in response to the H_2_O_2_ challenge. In accord, H_2_O_2_-exposure decreased cell survival in cooled HEK293 compared to normothermic cells (Fig. 2f).

Next, we reasoned that ROS scavenging enzymes may get depleted during cooling because of inhibition of their transcription and translation. To substantiate such hypothesis, we measured protein levels of the well-known mitochondrial scavenger manganese superoxide dismutase (MnSOD) in cooled HEK293 cells. Indeed, 6 h of hypothermia induced a clear decrease in MnSOD protein expression (Fig. 2g). To overcome cold induced lowering of endogenous scavenging, HEK293 cells were treated with the antioxidant trolox (a vitamin E analogue) prior to hypothermia, which effectively blocked the increase in MDA levels of cooled HEK293 cells (Fig. 2h).

## Discussion

In the present study, the effect of temperature (37–7 °C) on mitochondrial behavior was studied in whole kidneys, cells and isolated mitochondria. Our results demonstrate a substantial larger decrease in mitochondrial oxygen consumption than ROS production at hypothermic temperatures, accompanied with increased oxidative damage in hypothermia. The latest can be attributed to a decreased endogenous ROS scavenging in the cold.

In organ transplantation, core organ temperature undergoes various shifts during subsequent procurement, transportation, and transplantation of the graft, fluctuating between 1 and 37 °C [11]. The present study shows that a temperature drop from body temperature to room temperature induces a rapid decrease in mitochondrial activity. It can thus be suggested that the first cold flush in the donor, when organ temperature is decreased to 10–20 °C, already highly decreases oxygen consumption. Additional cooling, with the potential risk of activating damaging pathways, may be even detrimental.

The concept of cooling during preservation is largely based on the idea that the graft is maintained in a hypometabolic state, decreasing energy utilization during ischemia, and preserving essential mechanisms to generate ATP [13]. Indeed, we showed the oxygen consumption followed the Q_10_ line. Surprisingly, slowing down the electron transport chain by cooling reduces mitochondrial free radical production to a substantially lesser extent than O_2_ consumption, contrasting to the Q_10_. This is in accord with our own and other studies’ findings that the production of H_2_O_2_ is clearly present during cold preservation [14]. We found lower H_2_O_2_ production at hypothermic compared to normothermic temperatures. However, H_2_O_2_ production is relatively high when it is compared to the much stronger decrease in oxygen consumption and mitochondrial membrane potential (MMP) during hypothermia.

The relatively high ROS production and ensuing higher lipid peroxidation in cooled tissue and cells seems to originate from two processes. First, a difference in activity between electron chain complexes can be suggested. Even though the last complexes of the electron transport chain seem to be nearly inactive, given the low MMP and oxygen consumption, the first complexes can still be relatively more active. Or in other words, complex I and III, the complexes known to be the primary source of ROS, can have a relative less decrease in activity in hypothermia compared to complexes IV/V, inducing the production of free radicals [15, 16].

Secondly, besides ROS production, ROS scavenging plays an important role [17]. ROS damage can be seen as a result of a misbalance in ROS production and scavenging. Since the observed ROS production in hypothermia is relatively high, but lower compared to normothermia, the increased MDA levels at 7 °C can only be explained by a failure of scavenging. One of the underlying defense mechanisms by which mitochondria neutralize ROS is the superoxide radical converting enzyme MnSOD. MnSOD converts ROS to H_2_O_2_, and, in combination with catalase, H_2_O_2_ is further degraded to H_2_O [18, 19]. Indeed, exogenous H_2_O_2_ exposure resulted in more damage in hypothermic compared to normothermic HEK293 cells, suggesting an impaired catalase function. Thereby, we found decreased MnSOD levels during hypothermia, suggesting an impaired MnSOD function in the cold.

The proposed mechanism, i.e. that cooling lowers anti-oxidant capacity, is further substantiated by overruling the endogenous antioxidant function by exogenous antioxidant treatment. Indeed, treatment with the vitamin E analog trolox resulted in MDA levels comparable to baseline, even after exposure to hypothermia [20, 21]. In line with this, previous studies have shown favorable results after using mitochondrial targeted antioxidants in renal IRI models [22, 23], including the reduction in mitochondrial swelling during cold preservation [14, 24]. However, a significant challenge lies in the delivery of antioxidant enzymes to targeted sites [25]. It can be questioned whether a flush with anti-oxidant rich preservation solutions, such as UW, can deliver anti-oxidants in sufficient amounts to all mitochondria in a human sized kidney.

### Clinical significance and future directions

Most transplant organs worldwide are still preserved on ice with UW solution as the gold standard. As our results indicate, ROS still cause damage under hypothermic conditions, accentuating the importance of reducing oxidative stress during static cold storage (SCS). Various strategies to reduce oxidative stress during SCS of organs have been studied with good results (e.g. modified preservation solutions with antioxidants, treatment with hydrogen sulfide or immune targeting therapy) [26–29]. The widely used UW solution, for example, contains the antioxidants glutathione and allopurinol [8]. More recently, oxygenated HMP has been shown to improve graft function and attenuate mitochondrial dysfunction as evidenced by increased NADH and reduced ROS formation [7]. It has also been postulated that oxygenation of cells in the cold preserves mitochondria and limits ROS release [30]. The use of an oxygen carrier with anti-oxidant capacities (e.g. scavenging of ROS) for example, was shown to limited ROS-induced toxicity and reduce inflammation [31, 32]. Hyperoxia, on the other hand, can also induce excess ROS production. A tight balance between ROS and anti-ROS (or ATP) components remains key.

Donor preconditioning with catecholamines might also ameliorate cold preservation injury and improve outcome after kidney transplantation. As such, donor treatment with low-dose dopamine improved the kidney’s tolerance to accumulated ROS during cold ischemic storage and reduced the need for dialysis after transplantation [33, 34]. In vitro studies also demonstrated that catecholamines protect cells against cold preservation injury by scavenging of ROS or inhibition of ROS production [14, 35]. Compared to HMP, donor preconditioning with dopamine is associated with lower costs and workload to achieve improved outcome after kidney transplantation [36]. Donor preconditioning also offers the advantage that other organs from the treated donor also benefit from the intervention [37–39]. Therefore, donor pretreatment before an organ is exposed to cold ischemic injury provides an interesting tool to prevent the detrimental effects of ROS accumulation during hypothermia.

Cold organ preservation has also been studied in relation to hibernating animals. Interestingly, hibernating animals have shown to initiate safe metabolic suppression without organ damage when temperatures were around 4 °C [40, 41]. The phenomenon of shutting down most fueling processes and preserving mitochondria leads to survival of the animal after being in the cold for a long period of time. Even more, hibernating animals have shown to withstand iatrogenic damage outside the hibernation season [42–45]. Research from our center has shown that hibernator hamster kidney cells maintained MMP and ATP production, without an increase in oxygen radicals during cold preservation and rewarming [46]. Potentially by stabilizing the electron transport chain, reducing the discrepancy in activity between different mitochondrial complexes. In contrast, non-hibernator cells show loss of membrane potential, decreased ATP and increased MDA levels, similar to the results in this study [46]. Others have shown that lower MMP can be associated with an increased risk of delayed graft function after kidney transplantation [47]. In addition to hibernation, the effects of gasotransmitters H_2_S, CO and NO, have been shown to decrease ROS formation, potentially via mitochondria [48–50].

Although the present study describes important events during hypothermic preservation, we did not use a reoxygenation model. A recent study in vitro showed in a hypoxia-reoxygenation model that cell survival was improved after preservation by subnormothermic temperatures (19–32 °C) compared to 4 °C and 37 °C [11, 51]. However, in the ex vivo setting, hypothermic preservation tended to reduce damage more than subnormothermic temperatures. In a mouse model, experiments with solely cold ischemia showed detrimental kidney damage [52]. Yet, the most suitable preservation temperature depends on many factors and temperature optimization for kidney preservation will require more thorough investigation. The present study contributes to understanding the cellular processes that take place during hypothermic preservation, which can guide new approaches to improve post-transplant outcome.

## Conclusions

In conclusion, we show here that temperature highly affects maximum oxygen consumption rate of kidney mitochondria. This study also demonstrates that SCS brings about a substantial discrepancy between lowering of oxygen consumption versus radical production, while negatively affecting ROS scavenging capacity. Collectively, our data underline the importance of proper preservation of mitochondrial function and antioxidant treatment during preservation by cold storage. Hence, techniques such as oxygenated machine perfusion, donor preconditioning, using gasotransmitters, or inducing hibernation are promising tools to reduce the formation of ROS during preservation.

## Additional file

**Additional file 1: Figure S1.** A: Live kidney oxygen and temperature registration during whole kidney perfusion at different temperatures. B: Live kidney flow registration during whole kidney perfusion at different temperatures. **Figure S2.** Oxygen consumption versus temperature in whole kidney perfusion. Data shown for up and downwards temperature curve. The Q_10_ line was fitted using the R package respirometry. **Figure S3.** A: Amplex Red assay verification, RFU levels at different concentrations H_2_O_2_, at different temperatures over time. B: Full membrane for the western blot on MnSOD. Arrow at 25 kDa. C: Full membrane for the western blot on B-actin. Arrow at 50 kDa.

## Data Availability

The datasets used and/or analyzed during the current study are available from the corresponding author on reasonable request.

## Abbreviations

ATP: adenosine triphosphate
HMP: hypothermic machine perfusion
HEK293: human epithelial kidney cells
I/R: ischemia/reperfusion
MDA: malondialdehyde
MMP: mitochondrial membrane potential
MnSOD: manganese superoxide dismutase
NR: neutral red
ROS: reactive oxygen species
SCS: static cold storage
SEM: standard error of the mean
UW: University of Wisconsin
