# Supplementary material for: Renal temperature reduction progressively favors mitochondrial ROS production over respiration in hypothermic kidney preservation
Source: J Transl Med. 2019 Aug 13;17:265. doi: 10.1186/s12967-019-2013-1 (PMC6693148; doi:10.1186/s12967-019-2013-1)
Supplement: Supplementary file 1 — Additional file 1: Figure S1. A: Live kidney oxygen and temperature registration during whole kidney perfusion at different temperatures. B: Live kidney flow registration during whole kidney perfusion at different temperatures. Figure S2. Oxygen consumption versus temperature in whole kidney perfusion. Data shown for up and downwards temperature curve. The Q10 line was fitted using the R package respirometry. Figure S3. A: Amplex Red assay verification, RFU levels at different concentrations H2O2, at different temperatures over time. B: Full membrane for the western blot on MnSOD. Arrow at 25 kDa. C: Full membrane for the western blot on B-actin. Arrow at 50 kDa. [file 12967_2019_2013_MOESM1_ESM.docx]

**Additional information**

**Figure S1.** ***A:*** *Live kidney oxygen and temperature registration during whole kidney perfusion at different temperatures.* ***B:*** *Live kidney flow registration during whole kidney perfusion* *at different temperatures.*

*
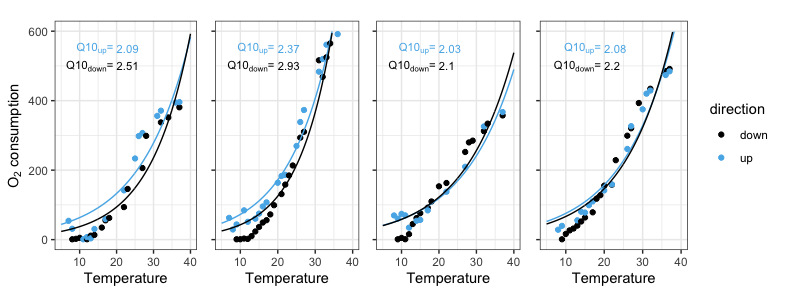
***Figure S2.** *Oxygen consumption versus temperature in whole kidney perfusion. Data shown for up and downwards temperature curve. The Q10 line was fitted using the R package respirometry.*

**Figure S3.** ***A:*** *Amplex Red assay verification, RFU levels at different concentrations H2O2, at different temperatures over time.* ***B:*** *Full membrane for the western blot on MnSOD. Arrow at 25 kDa.* ***C:*** *Full membrane for the western blot on B-actin. Arrow at 50 kDa*
